# Improving Efficiency and Quality of the Children’s ASD Diagnostic Pathway: Lessons Learned from Practice

**DOI:** 10.1007/s10803-017-3415-7

**Published:** 2017-11-30

**Authors:** Marion Rutherford, Morag Burns, Duncan Gray, Lynne Bremner, Sarah Clegg, Lucy Russell, Charlie Smith, Anne O’Hare

**Affiliations:** 1 School of Health Sciences, Queen Margaret University, Queen Margaret University Drive, Edinburgh, Scotland EH21 6UU UK; 2 NHS Lothian, Children’s Services, Royal Hospital for Sick Children, 5 Rillbank Terrace, Edinburgh, EH9 1LS UK; 3 Mental Health Access Improvement Team (MHAIST), Information Services Division (ISD), NHS Scotland, St Andrew’s House, Waterloo Place, Edinburgh, UK; 4 Child Life & Health, School of Clinical Sciences, University of Edinburgh, Edinburgh, UK

**Keywords:** Waiting times, Autism diagnostic assessment, Children, Pathways

## Abstract

The ‘autism diagnosis crisis’ and long waiting times for assessment are as yet unresolved, leading to undue stress and limiting access to effective support. There is therefore a significant need for evidence to support practitioners in the development of efficient services, delivering acceptable waiting times and effectively meeting guideline standards. This study reports statistically significant reductions in waiting times for autism diagnostic assessment following a children’s health service improvement programme. The average wait between referral and first appointment reduced from 14.2 to 10.4 weeks (t(21) = 4.3, p < 0.05) and between referral and diagnosis shared, reduced from 270 to 122.5 days, (t(20) = 5.5, p < 0.05). The proportion of girls identified increased from 5.6 to 2.7:1. Methods reported include: local improvement action planning; evidence based pathways; systematic clinical data gathering and a training plan. This is a highly significant finding for many health services wrestling with the challenges of demand and capacity for autism diagnosis and assessment.

## Introduction

Considerable stress is present for families and individuals with autism spectrum disorder (ASD), which is exacerbated through delayed or protracted diagnostic assessment (Crane et al. 2015). ASD is a heterogeneous condition and diagnostic assessment across the lifespan is in many ways more of an art than a science, involving the interpretation of intricate information from a variety of sources (Matson et al. 2012). In a nationalised health service, aiming to provide equitable public services, free at the point of delivery, this complexity contributes to the challenge of delivering timely and effective provision, within the resources available.

Despite high levels of adherence to evidence based clinical guidelines (McKenzie et al. 2016; National Institute for Health and Clinical Excellence (NICE) 2011; Scottish Intercollegiate Guidelines Network (SIGN) 2016) the average age of autism diagnosis for children has not reduced in the last 10 years, with the median age of diagnosis being 55 months (Brett et al. 2016). The mean age of diagnosis is recently reported as 62.8 months (Oswald et al. 2015); 89 months (Crane et al. 2015) and 105 months (Rutherford et al. submitted (b)) with outcome affected by age group being studied. The need to improve upon the delays in receiving assessment and diagnosis of ASD (Rutherford et al. 2016a; Wilson et al. 2013) and to understand how to adapt service delivery to meet the needs of this population is a research priority (Autistica 2015). Given the well documented challenge in translating research evidence into practice in healthcare, there is a need for clinical research partnerships to develop and evaluate solutions to this problem. Recent clinical research provides evidence based guidance to enable service providers to identify the focus for targeted intervention to address the challenge of reducing the wait for ASD assessment (Rutherford et al. submitted (a), 2016b) and we were interested in whether a clinical children’s service could apply this evidence to improve efficiency and reduce the duration of assessment, whilst maintaining a high quality service.

### Time Standards for Diagnostic Assessment

In order to target reductions in waiting times for assessment, health services need to know what length of wait is expected (Holzer et al. 2006) and whilst there has not been an international push to set time standards, the longstanding and not otherwise contested UK recommended time scale from referral to diagnosis in children in the National Autism Plan for Children (NAP-C) is 119 days (Le Couteur 2003).

For highly complex cases or where a second opinion is required from a tertiary service, the NAP-C recommended duration from the start of the specialist assessment to conclusion is a further 6–8 weeks (42–56 days), taking the maximum recommended duration of assessment from referral to diagnosis for the most complex cases to 175 days (LeCouteur 2003). Previous research identified that many ASD diagnostic services do not have a means of recording waiting times and for those that do, few meet the NAP-C 119 day standard (Palmer et al. 2010). To date, very few studies have reported on either the duration of the diagnostic assessment process, how to define “complexity” or results of interventions to reduce this duration in child health services.

The jury is still out on how we set the bar for who is so complex that they need to see a highly specialised team and who can be reliably diagnosed by a well-trained local team. However this seems to be an important factor in setting expectations for families about optimal duration of the process. McClure et al. (2010) found that following a programme of training, there was a high degree of consistency between local and specialist teams. Historical practice of ASD diagnostic assessment taking place via a small number of specialists was replaced by a new approach, made necessary given the increasing recognition of ASD and demand on health services. Through broadening the skill set of community teams in ASD assessment, they made significant reductions in waiting times, from 36 to 13 weeks.

More recently, the NICE (2016) quality statement on autism in children advises no longer than 3 months (12 weeks) between referral and first assessment with no further guidance on duration of assessment. In a recent national study from Scotland (McKenzie et al. 2015), the average wait from referral to first assessment was 200 days (SD 209, range 0–1172 days) and the total wait from referral to diagnosis in child health services was 332 days (SD 319, range 0–1942 days) with 74% of child cases exceeding the 119 day standard. Although it is acknowledged that more complex cases are expected to take longer, evidence suggested that clinicians within health services do not explicitly adapt pathways and approaches for the needs of more or less complex cases (Rutherford et al. 2016c), for example by gathering relevant information before the first specialist appointment (McKenzie et al. 2015).

Rutherford et al. (submitted (a)) found that it was possible to significantly reduce the waits for autism diagnosis in adult health services (these were the waits between referral and first appointment and between first appointment and diagnosis) through implementing a 12 month programme of service change together with ASD specific approaches. The authors suggest that increased waiting times may be due to increased clinical complexity in some cases, and propose action plans to better manage services through: a focus on the adoption of evidence based practice as advised in clinical guidelines; informed use of standardised instruments; establishing clear pathways which detail constructive use of time and tools at each stage from recognition through to diagnosis for more or less complex cases; consideration of training needs; using structured processes and proformas to gather diagnostic information and avoid duplication of effort and a planned change programme which takes account of local context.

The rise in waiting times is also likely to be linked to the widely accepted increase in recognition of ASD and increasing demand on services for diagnostic assessment (Centers for Disease Control and Prevention 2012), adding further evidence of the need for efficient practice to meet this demand.

### Components of Service Improvement

Healthcare interventions to improve service provision are highly complex (Pawson et al. 2014) with a dynamic range of solutions applied, from strategic and organisational change, to role change or procedural, administrative and motivational change. All of which must take account of the local context as well as the evidence base. The following components of planned service improvement in ASD diagnostic services arise in the literature.

### The Importance of Good Data

Good quality health data is accurate, consistent, fit for purpose, reliable and timely and such data is essential to accountability in health care (Requejo et al. 2015). However in UK ASD health services, data collection practice does not routinely meet these criteria. It is very hard to know what improvement is required in local areas because many do not have an ASD diagnostic assessment pathway. Even basic information, such as the number of referrals, or length of assessment is not routinely collected (Palmer et al. 2010). For health service teams to apply research evidence and evidence based guidelines, the first step may be to establish good quality and efficient processes for data collection, to inform service planning.

### ASD Pathways

**Fig. 1:**
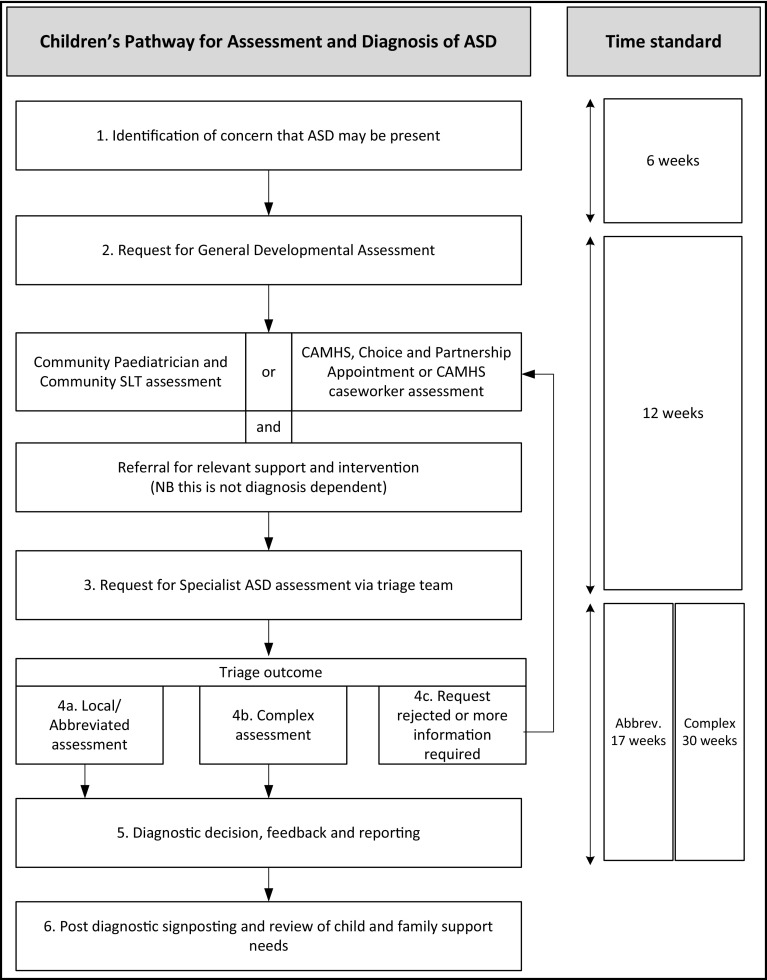
Children’s pathway for assessment and diagnosis of ASD

Following the publication of the NICE (2011) clinical guideline, clinicians can access related resources with regard to ASD pathways. Rutherford et al. (2016b) provide a review of core elements of care pathways as a complex intervention to support more efficient and effective ASD diagnostic assessment. The ASD pathway developed in the current study (see Fig. 1) was based on evidence of processes and practice that make a difference to service waiting times, including: recognition of the value of dialogue with clinicians in the development of the pathway and taking account of their views and experiences to support acceptance and compliance with the new pathway (Rutherford et al. 2016b); the value of good quality data to support accountability and planning (Requejo et al. 2015); the knowledge that adherence to clinical guidelines is unlikely to increase waiting times (McKenzie et al. 2016); the importance of a clear plan and process, with timely collection of information and a plan based within a theoretical framework to implement service improvement (Rutherford et al. submitted (a); Melton et al. 2012). For the intervention reported here, the pathway (Fig. 1) was shared with clinicians, together with more detailed pathway guidance to inform them about processes, resources available and standards at each stage in the pathway. This is available from authors on request.

### Flexible Approaches to Assessment

The pathway guidance recommends that all assessments should include the core components of developmental history, contextual assessment and direct observation. Aspects of this assessment may be completed during the general developmental assessment phase or during specialist assessment. There are some children with ASD, where the ASD signs and symptoms are easily apparent during general developmental assessment (stage 2 of the pathway) or at the point of triage (stage 3 of the pathway). It is proposed that these cases follow an “abbreviated pathway” without onward referral to a specialist team. Using reports and observation made by the local community staff, in a range of contexts, the ASD diagnosis is confirmed by two or more clinicians, through mapping evidence from assessments to DSM 5 criteria (American Psychiatric Association 2013). A member of the local team, who has a relationship with the family, will share the diagnosis and provide literature and signposting.

Other cases are complex (NICE 2011) because of within child factors (such as co-morbidity) or contextual factors (such as co-occurring neglect or family/professional disagreement). A perception of complexity may also arise where a local team feels this is a complex case for them because of a misfit between staff skill set and familiarity with children of a particular age and developmental stage or children with particular differential diagnoses to consider. For these reasons Autism ACHIEVE Alliance (AAA) research describing complexity factors within a clinical population (Rutherford et al. submitted (b)) has suggested, that one size does not fit all. Standardised instruments or referral to specialist assessment by expert level practitioners may be needed for complex cases but not for all. Flexible but informed use of standardised instruments is recommended (Rutherford et al. 2016c).

### Referral Management and Triage

A range of children’s community health services use triage to screen for inappropriate referrals and to support the most appropriate next steps being taken (Curran et al. 2015). In Child and adolescent Mental Health Services (CAMHS), the Choice and Partnership approach (CAPA) uses a recommended procedure: children/families newly referred to CAMHS are invited to an initial face-to-face ‘Choice appointment’ which aims to reach a shared understanding of needs. This occurs in place of an assessment. A range of options can then be offered, including signposting to other services, self-help strategies or appropriate specialist interventions. If the child and family choose to be seen for further appointments within the service, then they are invited to book ‘Partnership appointments’ to work on mutually agreed targets (Robotham et al. 2010). CAPA is widely used as a form of triage to manage long waiting lists through managing non-attendance, efficient resource allocation and prioritisation of most needy cases (Naughton et al. 2015). Children with ASD may not benefit from comparisons for prioritisation with children with life threatening mental health presentations. In a climate which encourages early identification and a preventative approach to potential challenges in ASD, a specific ASD triage may be helpful. Through recognition that there is a regular demand for ASD assessment, capacity for this can be planned. Multi-disciplinary ASD triage with experienced staff from CAMHS, Community Child Health (CCH) and Speech and Language Therapy (SLT), allows allocation of cases to abbreviated or complex pathways at the outset. This avoids the challenge of double waiting lists between local generalist and centralised specialist health services. Complex cases do not have to go through the abbreviated team first.

### Service Configuration

There is very little evidence to guide optimal service configuration, however there is consensus that core multi-disciplinary diagnostic teams for children are advisable and should include representation from Speech and Language Therapy (SLT), Clinical Psychology, Psychiatry and Paediatricians (NICE 2011; Rogers et al. 2016; SIGN 2016). AAA Research identified that younger children tended to go to Joint CCH and SLT services whilst older children went to CAMHS, with only one in eight services in the AAA study configured with all three professional teams. It is anticipated that with use of a single pathway and guidance, consistent expectations between services can be engendered, avoiding duplication of waits between different services in the pathway, which are operating to the wider demands of their own service. This duplication can occur when a child waits to see a local diagnostic team who then see them and decide they are too complex. The child then waits again to see the specialist team, who do very similar assessments but have a different skill mix or level of skill. Duplication can also occur when a child waits to see one service (e.g. CAMHS) and then it is decided that the assessment cannot be progressed until they see another service (e.g. SLT) and the child waits again. Early triage to consider complexity and skill mix required in the team can reduce such duplication and thus shorten waiting times.

### Training and Mentoring of Staff

Effective training can impact positively on practices which improve quality and efficiency of ASD diagnostic services (Hathorn et al. 2014; McClure et al. 2010) and having limited staff with specialised training can impact negatively (Duffy et al. 2002). The National Health Service (NHS) Education for Scotland (NES) Autism Training framework (2014) was devised following review of available published evidence, clinical guidelines and local consultation. The framework permits mapping of staff skills and knowledge for ASD diagnostic assessment at 4 levels (informed, skilled, enhanced and expert practice levels). The framework can be used by individuals, organisations or training providers to identify current or future training needs and levels. Detailed descriptions of essential knowledge at each level are outlined in the framework and levels of skill required by different staff depend on the nature, extent and likely impact of daily contact with individuals with ASD, rather than defining levels specific to profession or position in a service. The levels are summarised as follows using descriptions from the framework: Informed level describes the requirements for all staff working in health and social care settings, in relation to ASD. Skilled level is required for all staff with direct and/or frequent contact with individuals on the spectrum or those who have a role with high impact on those individuals. Enhanced level is for practitioners with more regular or intense contact with individuals with ASD where their role focuses specifically on autism, provides specific ASD interventions or manages the service for individuals on the spectrum. The Expert level describes the highly specialist knowledge and skills required by practitioners who, have a specialist role in the care, management and support of people with ASD. It is not expected that staff at informed or skilled level undertake or lead on diagnostic assessment but they may contribute information to the process.

In the past ASD diagnosis was largely undertaken by a small number of expert practitioners, however the growing recognition of ASD as one of the commonest developmental disorders, means that health staff working with children with developmental delay, speech, language and communication impairments and mental health difficulties at all levels will come into regular contact with individuals with ASD and therefore need skills at the enhanced level. Increasingly front line community staff have acquired skills in assessment of children with ASD and therefore, when such staff do have enhanced or even expert skills, diagnosis can take place locally, with less duplication. An understanding of the current skills and training needs of staff was a core component of the improvement plan.

### Aims

This study aims to

- Identify the baseline number of referrals and duration of ASD diagnostic assessment for children (aged 0–18) across a health board before a single evidence based ASD care pathway was in place
- Describe the pathway development process and service changes implemented
- Evaluate the effects of the new pathway for ASD diagnosis on knowledge of service demand, duration of assessment and waiting time.

## Method

### Governance Processes

The local (NHS) Research and Development Department and the NHS Quality Improvement Team granted approval for this study.

### Participating Health Services

There are 14 health boards across Scotland and this study takes place in one of these, with a population of 850,000 representing 16% of the Scottish population. In a prevalence study, in the same locality, 75% of children with ASD were identified (Harrison et al. 2006). Previous unpublished local data identified that between 25 and 50% of initial referrals to SLT clinics and 25% of CCH referrals stated concern over ASD, indicating high demand within each service. Within this health board, there are four local authority areas (labelled as A–D throughout the study) with area A consisting of 50% of the population, Area D consisting of 25% and Areas B and C making up the other 25%. Across all areas, seven separate local teams were identified (teams 1–7). Each team operated independently in the management of referrals, although there were some shared protocols across teams. Traditionally CCH and SLT teams worked jointly as one team, CAMHS operated as a different team, with both groups undertaking ASD diagnostic assessment separately. There were 3 teams in Area A, 2 CAMHS teams and one CCH/SLT team; in Area B there was a single joint CCH/CAMHS/SLT ASD team; in Area C, there were 2 teams, one CCH/SLT and CAMHS team; and in area D there was one CCH/SLT team (with no CAMHS team). Therefore 7 separate teams offering ASD diagnostic assessment, with different waiting times were included in the baseline measures. A new CAMHS team was additionally included in the post implementation data.

### Ethnicity

This locality serves a largely Caucasian population, with 25% percent of children from black or minority ethnic (BME) backgrounds and 10% with English as an additional language (EAL). Individual ethnicity data were not collected.

### Procedures

**Fig. 2:**
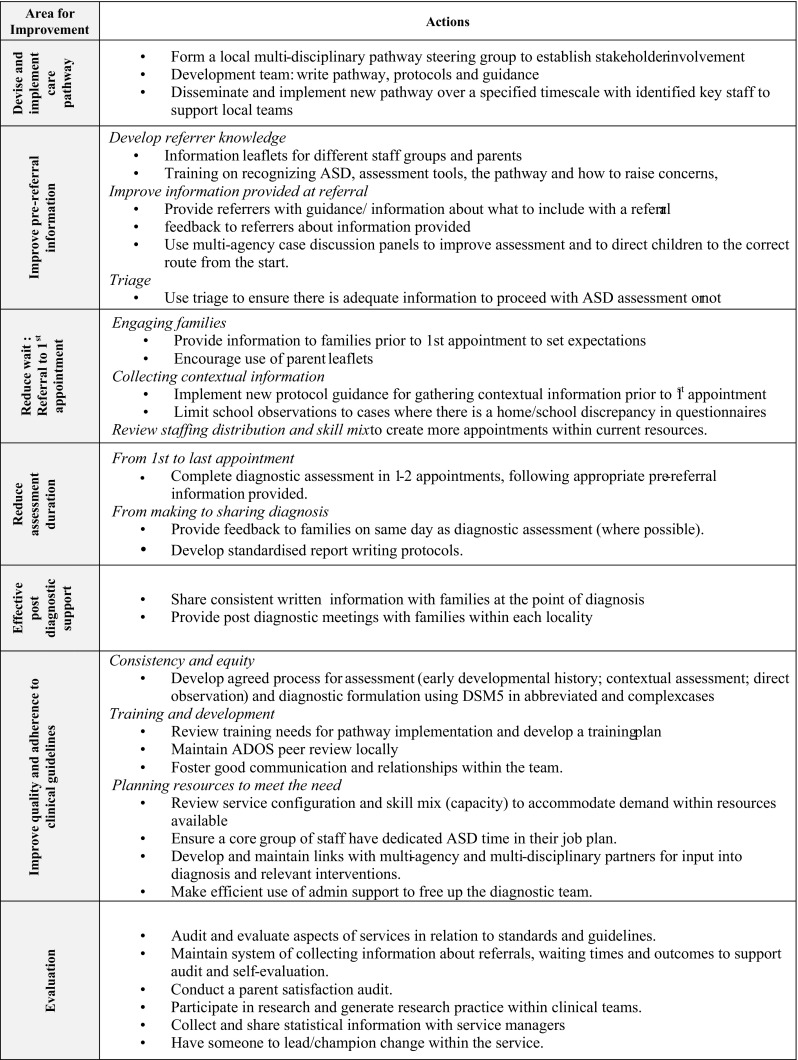
Local action plan for the ASD diagnostic assessment service

The work reported comprised several steps: (a) baseline information gathering about current practice and national guidance; (b) development of an action plan (Fig. 2); (c) writing and achieving consensus to implement the new pathway (d) setting up a clinical database for recording and measuring involvement in the pathway for each child referred (e) statistical analysis of the data. Two clinicians were seconded for 1 day a week for a year to undertake evaluation and development work reported. They were supervised by clinician researchers from Medical Paediatrics and SLT and supported by a multi-disciplinary steering group.

### Baseline

We used two methods to gather baseline data.

#### Staff Consultation

Evaluators met with local diagnosing teams to review current practice across the health board area. Specifically, CCH led teams in each area and CAMHS led teams in three of four areas. One clinician from each team (n = 7) was interviewed about main aspects of the diagnostic services such as personnel involved and process followed.

#### Case Note Analysis

At baseline, teams 1–7 did not follow a consistent data collection process for children referred for ASD assessment. Data were collected in a systematic way in three out of seven teams. These teams kept written or electronic lists of children referred for ASD assessment, together with dates of referral. For four of seven teams (2 from area A, 1 each from areas B and C), ASD referrals were not kept in a list or counted as separate from the general list of referrals for all types of assessment.

We were therefore only able to estimate the annual number of referrals at this point. Retrospective case note analysis was undertaken (n = 79) using a data gathering tool adapted from the AAA Individual Data Collection tool (McKenzie et al. 2015). This bespoke, non-standardised tool was developed through consensus between expert practitioners for the specific purpose of data collection in this study. It was used to identify wait times across the different teams in this health board area. Data collected using this tool included: key demographic information; dates of referrals; dates of key time points along the pathway and whether a diagnosis of ASD was given or not.

### The Local Action Plan

Evidence gathered was synthesised to form the Local Action Plan (Fig. 2) to serve as a focus for the range of actions required to implement this complex health service change. Seven key areas for improvement were identified: (1) devising and implementing a care pathway; (2) Improving information gathered and shared at the pre-referral stage; (3) Reducing the wait between referral being accepted at triage and first appointment; (4) reducing the assessment duration at different stages in the pathway; (5) providing effective post-diagnostic support; (6) improvements in quality and adherence to ASD clinical guidelines and (7) Evaluation to support ongoing review and reflection.

### Pathway

Evidence was used to write the *pathway for assessment and diagnosis of children with ASD* (Fig. 1) together with evidence based guidance. Once written, agreement to follow the new pathway was obtained from all stakeholders and the pathway was disseminated as a written document to all relevant teams and through a series of single profession and multi-disciplinary face to face events over the course of 2015. A pathway steering group was established, meeting three times per year, to monitor and review the pathway. By 2016, all 4 areas were implementing the new pathway with monthly triage meetings in place.

### Data Collection

A database was created to support the new pathway processes. Data was collected for each child referred for ASD assessment, using the data collection tool and was undertaken as part of routine clinical care, by clinicians and administrative staff.

### Data Analysis

Data reported in this study was analysed for cases referred in a 12 month period (from the second year of pathway implementation), once practice was becoming established.

### Training and Mentoring

An evaluation of current skills and training needs was undertaken through a staff survey based on the NES Autism Training framework (n = 100). A review of the framework identified that the lowest optimal level of skills and knowledge for clinicians undertaking assessments which contribute to ASD diagnosis was the Autism Skilled level. This survey results (available in an unpublished report) were used to develop a training plan to accompany the pathway.

## Baseline Results

### Baseline Results of Interview with Clinician from Each Service

Wide variation in practice was identified. There were differences in referral criteria. For CCH, if the child had been seen recently at CAMHS or had an entirely behavioural presentation then a referral would not be accepted, otherwise the only criteria was the child presents with difficulties that may be ASD after general developmental assessment. Service configuration also varied. Within CCH the diagnostic teams typically consisted of a paediatrician and specialist or highly specialised SLT. The CAMHS diagnostic team was more varied including a combination of psychiatrists, clinical psychologists, community mental health workers, occupational therapists (OTs), social workers and nurses.

Information gathered for diagnosis was generally similar, with all teams having staff trained in use of the ADOS—Autism Diagnostic Observation Schedule (Lord et al. 2000). However the tools used differed. For example one team used the Adolescent Autism Spectrum Quotient (Baron-Cohen et al. 2006) and the Australian Scale for Aspergers (Garnett and Attwood 1998) to gather contextual information from schools whereas half the teams typically used the Social Responsiveness Scale (SRS) (Constantino and Gruber 2012). This is reasonable as no specific tools for gathering contextual information are specified by guidelines but empirically some have greater construct validity and standardisation. The timing of when information was gathered varied, for example, CAMHS normally included a classroom observation early in the process. However, CCH/SLT teams would only do this if the information from the standardised contextual assessment questionnaires was discrepant between parents, educators, or other agencies who know child’s presentation and was not in keeping with the clinical observation. This variability meant the client journey from 1st appointment to diagnosis varied significantly across teams, in terms of: the information gathered prior to referral for ASD assessment; the number of different contacts with professionals; the number and type of professionals involved and the types of assessments completed. Each of these factors has the potential to affect duration of assessment and waiting times.

### Baseline Waiting Times and Referral Rates

Of the 7 teams, 4 had no mechanism for recording waiting times. For those clinics who were able to supply this information none were meeting the targeted timescales. For those with data, the average waiting time for an initial appointment for ASD diagnosis was reported to be just over 6 months.

The teams estimated that across the health board area, there were 340 referrals in the previous 12 month period in 2013–2014.

### Baseline Case Note Analysis

From all 7 teams undertaking ASD diagnostic assessment, 79 cases were audited. The size of the sample represents the relative populations served by each team. The duration of WAIT 1 (time between referral for specialist assessment and first assessment appointment) and WAIT 2 (time between referral for specialist assessment and diagnosis shared) were measured. In the sample, the average age of diagnosis was 82 months (6; 10 years), with a standard deviation of 45 and the range was 18–214 months (17; 10 years). There were 67 boys (85%) and 12 girls (15%), a ratio of 5.6:1. The average duration across all teams was 270.2 days (Table 1).

**Table 1:** Baseline assessment of waiting times

Local authority area	Service	Number of cases audited	Mean duration from referral to diagnosis shared (days)
A	1 CCH	24	255.7
A	2 CAMHS	8	282.4
A	3 CAMHS	6	314.4
B	4 CCH and CAMHS	10	139.9
C	5 CCH	10	232
C	6 CAMHS	8	305.25
D	7 CCH	13	361.6
Total		79	All Health Board mean duration 270.2 days (38.6 weeks)

### Pathway: Key Changes

The outcome of the literature review and local scoping exercise led to the development of the new pathway. As well as being consistent with evidence based clinical guidelines, key features and new consensus included: a single pathway; a single way of recording referrals; a triage process; use of the abbreviated/complex pathway; consensus on contextual assessment guidance; report writing guidance and information leaflets about the pathway for staff and families. Additionally alongside the pathway we undertook a *Training and Mentoring Project* to support implementation through assessment of training needs and delivery of core training.

## Results Following Implementation

### Referral Rate

All teams added new referrals to the database, with the exception of team 2 and therefore the 2016 total recorded referral rate continues to be an underestimate. The reason for non-compliance is lack of allocation of administrative support for this purpose. Although previous evaluation highlighted the inaccuracy of estimation, this service estimated by counting the number of ADOS assessments completed in 2016 and estimated around 100 cases were not on the database. Results showed that the actual referral rate (plus 1/8 teams providing estimated and not actual data) was 811 per year; more than double the original estimated referral rate. The actual number of referrals recorded in each area was as follows: Area A, n = 303; Area B, n = 86; Area C, n = 112 and Area D, n = 201. The new system permitted recording of the increase from 340 referrals per year to 711 and monthly referral numbers recorded have remained steady for the past 12 months.

### Gender Distribution

Information about gender of referrals (see Table 2) was only consistently recorded in one locality (representing 43% of referrals). In 2016, there were 259 referrals with completed assessments. Gender and positive ASD diagnosis were recorded for 70/85 males, 34/41 females. A diagnosis of “Not ASD” was given to 7/41 females and 14/85 males. In 2 cases no diagnosis was recorded and in the remaining 116 cases gender was not recorded. The ratio of males to females was 2.7:1 for referred and diagnosed cases.

**Table 2:** Age group distribution and gender of 2016 referrals

Age group distribution of referrals							
Age group (years)	A	B	C	D	Total number	Total percentage	
0–4	84	14	29	31	158	22.2	
4–8	114	29	29	62	234	32.9	
8–12	67	20	30	60	177	24.9	
12–16	33	15	19	45	112	15.8	
16–20	5	8	5	12	30	4.2	
Grand total	303	86	112	210	711	100	

Gender of referrals							
Gender	A	B	C	D	Total number	Assessments completed	ASD diagnosis
Female	73			5	78	41	34
Male	210			1	211	85	70
Missing data	20	86	112	204	422	133	118
Grand total	303	86	112	210	711	259	222

### Age of Referral

The total mean age of referral in 2016 was 7.9 years (range 1.4–8.0 years); the median age of referral was 7.1 years (85.2 months) and the majority (54%, n = 392) of referrals are under the age of 8 years. Table 2 presents the age groups of children referred, with 22.2% (n = 158) being children 0–4 years; 32.9% (n = 234) aged 4–8 years; 24.9% (n = 177) aged 8–12 years; 15.8% aged 12–16 years (n = 112) and only 4.2% (n = 30) between 16 and 20 years.

### Triage Outcomes

Triage outcomes are reported in Table 3. Of those referred 2.3% (n = 16) had already been given the diagnosis locally but were still added to the database and 11.3% (n = 80) were allocated to an abbreviated pathway. The majority of cases were allocated to complex assessment with 60% (n = 426) being allocated their next appointment with one or more relevant professional group (CAMHS, SLT and CCH) and a further 12.2% (n = 87) being allocated to an ADOS assessment as the next (complex) step following triage. The number receiving ADOS assessment after their first appointment was not collected but it should be stated that a proportion have this outcome as part of their assessment. For a small number, more information was requested or the referral was not accepted and they were removed from the list.

**Table 3:** Triage outcomes

Triage outcomes: number and percentage of referrals allocated to each initial triage outcome						
Outcome at end 2016	Assessment completed		Assessment not completed		Grand total	
	No.	%	No.	%	No.	%
Abbreviated	50	19.3	30	6.6	80	11.3
Diagnosis made pre-triage	3	1.2	17	2.9	16	2.3
Complex	156	60.3	266	59.7	426	60.0
ADOS	44	17.0	43	9.5	87	12.2
			Accepted at triage		609	85.8
More information requested	0	0.0	13	2.9	13	1.8
Removed from list	0	0.0	3	0.7	3	0.4
			Not accepted		16	2.2
Missing data (blank)	6	2.3	80	17.7	86	12.1
Grand total	259		452		711	100

### Diagnostic Rate

For cases where referral was accepted and the diagnosis was completed within the time being studied, we counted the number and percentage of cases where a positive ASD diagnosis was given and the number where the team concluded that the child did not have ASD. In the whole sample 84.6% (n = 219) received a positive diagnosis and 14.3% (n = 37) went through the assessment process with the conclusion that they did not have ASD (Table 4).

**Table 4:** Diagnostic rate: number and percentage referred who are diagnosed with ASD

Diagnostic rate for 2016 referrals: number and percentage where an ASD diagnosis was made (of 259 completed assessments)										
Outcome	A		B		C		D		Grand total	
	No.	%	No.	%	No.	%	No.	%	No.	%
Not ASD	19	15.4	5	13.9	5	22.7	8	10.3	37	14.3
ASD diagnosed	102	82.9	30	83.3	17	77.3	70	89.7	219	84.6
Incomplete/missing data	2	1.6	1	2.8		0.0		0.0	3	1.2
Grand total	123		36		22		78		259	

### Waiting Times

Statistically significant reductions in waiting times for assessment were identified (*p* = < 0.05), with the service meeting NICE and NAP-C standards following implementation of new working practices. Waits were measured in two ways. Table 5 outlines WAIT 1—the time between referral and first appointment. The average wait across this health service from January to July was 10.4 weeks, which compares favourably with NICE (2016) recommendations that this is < 12 weeks. We compared WAIT 1 in 2015 and 2016, using two-sample t-test (assuming unequal variances). The average wait times were found to be significantly different (*t*(21) = 4.3, *p* = < 0.05).

**Table 5:** WAIT 1: Wait from referral accepted to first appointment

Referral to first appointment average wait time in 2016 (for completed assessments) in weeks					
2015	A	B	C	D	Grand total
Jan	8.2			21.1	16.5
Feb	7.8			30.5	15.8
Mar	10.5			15.7	12.2
Apr	13.2			22.6	17.2
May	13.2			15.2	13.6
Jun	14.7			23.8	17.3
Jul	14.5			21.5	17.6
Aug	15.1			13.6	14.9
Sep	14.5			9.7	13.0
Oct	11.8			10.8	11.4
Nov	9.0			16.4	11.2
Dec	8.7			25.2	10.2
Average across health board area for available data 2015					14.2 weeks

2016	A	B	C	D	Grand total
Jan	11.6		6.4	8.1	10.5
Feb	14.6		1.0	1.2	12.4
Mar	11.4		10.1	7.9	10.4
Apr	13.9		7.5	6.6	10.0
May	10.0		6.4	8.5	8.5
Jun	11.9		5.0	6.9	9.0
Jul	16.1		9.8	7.1	11.8
Aug	11.7			7.5	9.1
Sep	12.0				12.0
Oct	6.8				6.8
Nov					
Dec				2.9	2.9
Average across health board area from January to July 2016					10.4 weeks

Data were collated and analysed in January 2017 and it may be helpful to explain why there is some missing waiting time data in Tables 5 and 6. Initially, there was a graduated transition for each service to start implementation and data collection. This is the reason for missing data in 2015. By 2016, compliance with the pathway and data collection was good, as shown by waiting time data in January-July 2016. Cases referred in August–December 2016 were still at an early stage in the pathway and therefore number of completed assessments and waiting time data points is small.

**Table 6:** WAIT 2: Wait from referral for specialist assessment to diagnosis shared

Referral to diagnosis shared: average wait time in 2015 and 2016 (for completed assessments) in weeks					
2015	A	B	C	D	Monthly average
Jan	29.1			22.6	24.7
Feb	34.2			35.8	34.8
Mar	32.2			34.0	32.8
Apr	30.8			28.1	29.6
May	30.9			20.4	28.8
Jun	26.4			25.9	26.3
Jul	30.0			29.2	29.7
Aug	24.6			15.9	23.3
Sep	22.8			16.9	21.5
Oct	17.3			16.0	16.9
Nov	25.5	29.9		21.2	24.8
Dec	23.4	24.9		35.5	24.4
Average across health board area for available data 2015					26.4 weeks

2016	A	B	C	D	Monthly average
Jan	22.2	24.4	18.8	15.5	20.9
Feb	24.4	23.0	11.7	11.0	22.4
Mar	18.4	22.2	25.1	14.4	18.7
Apr	18.9	23.2	28.9	9.5	17.2
May	18.1	28.1	14.7	11.7	15.7
Jun	17.9	10.1	10.9	10.9	13.7
Jul	19.8	17.3	15.4	13.6	16.7
Aug	18.0	12.6		12.8	13.9
Sep	12.8			7.0	11.7
Oct	10.0	11.1			10.5
Nov					
Dec					
Average across health board area, January to October 2016	20.0	21.1	16.7	12.1	17.5 weeks

Secondly we were interested in WAIT 2: time from referral to diagnosis shared, which NAP-C (2003) recommends is < 17 weeks. We compared the wait time between referral and diagnosis shared for available data from 2015 to 2016, across this health board using two-sample t-test (assuming unequal variances). The reduction in average wait times was found to be significantly different (*t*(20) = 5.5, *p* = < 0.05).

**Fig. 3:**
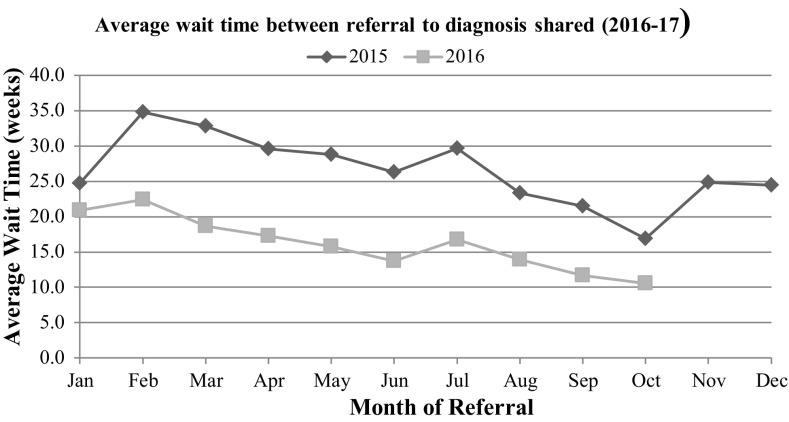
Average wait time between referral and diagnosis shared

Table 6, shows the gradual reduction in this wait time from 2015 and then from January to October 2016 and the finding that the mean wait for diagnosis using this measure was 17.5 weeks. As above, the missing data from November to December 2016 is explained by timing of data analysis 1–2 months later, when cases referred had not progressed far through the pathway. Figure 3 represents the downward trend and includes a comparison with 2015 data, showing this trend seems to be genuine.

### Training and Mentoring Outcomes

The survey sent to all CCH, CAMHS and SLT staff found that there was no one group who felt they had full knowledge in any of the identified areas of the NES autism training framework (NHS Education for Scotland 2014). Staff rated themselves at different levels across different competencies and a range of training needs were identified at each level. A training and mentoring portfolio tool was piloted that outlines knowledge and skills levels required for the *autism skilled, autism enhanced and autism expert levels*.

Information leaflets for universal services including GPs, Health Visitors, nurseries and schools were written which outline the ASD pathway and referral routes. These give referrers guidance on how to recognise ASD, what information they should gather, how to refer and what to expect following a referral. These leaflets are intended to be used alongside training for professionals working at the *informed* and *skilled* levels. At *skilled* level, uni-professional knowledge and skill requirements were summarised with signposting to e-learning modules. At *enhanced* level, a training day for 90 delegates was organised, covering core components of ASD assessment and diagnosis. The recordings and slides are available on the intranet of this health board as a permanent resource. At the *enhanced* level, a mentoring portfolio was produced and is intended for use in addition to training days. Practitioners are supported by a more experienced clinician within the diagnostic team. The portfolio consists of suggested activities to observe (e.g. parent interview); written examples (e.g. a feedback report) and opportunities for self and peer reflection on practice in using skills observed. For the smaller number of clinicians at *expert* level, there is a system of ADOS peer review. The steering group are exploring ways of increasing skills for other standardised tools including the ADI-R.

### Service User Feedback

Parental feedback on the assessment process was gathered via telephone interview with a small number of families (n = 7) at time of publication. Key themes were identified and the general feedback about the assessment experience was positive. Issues around post diagnostic support were raised. Further service user feedback will continue to be sought to evaluate the impact of the pathway.

## Discussion

The aim of the present study was to examine the effect of an evidence based pathway for diagnostic assessment of ASD in reducing the wait time between referral and sharing the outcome of ASD diagnostic assessment in a children’s service delivered jointly by CAMHS, CCH and SLT.

### Referral Rates

The study identified that without a systematic process for collecting data, the demand for ASD diagnostic assessment was vastly underestimated (by over 50%). In 2014 the estimated number of referrals was 340 and in 2016 it was found to be 711 with 7/8 teams providing this data consistently, with a further 100 estimated from 1/8 teams. This finding has significant implications in relation to accurate service planning for capacity to meet demand using service improvement methodology (NHS Scotland 2017; Scottish Government 2010). Although ongoing challenges with full compliance in data collecting by clinical teams presents an ongoing issue, the study has provided more accurate evidence of the minimum number of children being referred each year and the fact that this now remains steady suggests a good degree of accuracy. Clinicians will benefit from support of data management specialists to identify an effective way of gathering this information and we recommend that a national approach is developed in order to budget effectively and manage the need for ASD health services. Referral rates over 2016 have remained steady suggesting that we can plan to meet this demand with greater confidence than in the past.

### Gender and Age Distribution

Where this data was collected, there were more boys referred than girls, which is in keeping with international consensus (Rutherford et al. 2016a). However the reducing ratio of boys to girls of 2.7:1 (from 5.6:1 at baseline) could be indicative of a trend towards greater recognition of girls, supported by awareness raising as outlined in the local action plan (Fig. 2).

At baseline, the mean age of referral was lower at 6;10 years and after implementation this had risen to 7.9 years (93 months) which may be explained by the greater recording of CAMHS cases. However this is still broadly in keeping with other studies. The median age was 7.1 years (85.2 months), which is higher than reported findings from a range of studies over the last 10 years (Brett et al. 2016). It is not unexpected that 54% of referrals were under the age of 8 years and that number in the over 12 age groups, were smaller. Further work to support earlier recognition, through awareness raising and training of referrers is indicated.

### Benefits of Pathway Implementation

The pathway has improved consistency of practice. Prior to implementation there was a great deal of inconsistency, identified in the baseline interviews with clinicians and the case note analysis. Differences were identified in relation to time taken to complete an assessment, referral practice, number of contacts taken to make an assessment, the nature of the assessment undertaken and the reporting of information. It is likely that this would be confusing for families and non-health staff also working to meet the children’s needs. No leaflets or information were available to explain the referral and diagnostic assessment expectations. Following the implementation of the pathway there is now clear and available guidance for staff and information leaflets for families. Further evaluation of patient and staff experience and understanding of the pathway would be beneficial.

### Diagnostic Rate

Of those accepted at triage and for whom assessment was completed, 86% were given a diagnosis of ASD. Our interpretation of this is positive evidence of the effectiveness of the pathway which includes triage and referral management, a flexible approach to abbreviated and complex assessments, as well as very clear guidance and training for staff. As a result, there are proportionately fewer children who go through the full process, who do not have ASD. This is important in reducing the risk of unnecessarily raising parental concern over ASD when it is not present (Hedley et al. 2016). We have found relatively few studies with which to compare our findings on clinical diagnostic rate; McClure et al. (2010) reported that only 17/38 children assessed (45%) received an ASD diagnosis.

### Waiting Times from Referral to First Appointment

In contrast to previously reported waiting times (McKenzie et al. 2015), the evidence reported indicates that this service is able to meet the NICE (2016) standard for 3 months between referral for specialist assessment (following general developmental assessment (GDA) and first appointment). Our results provide firm evidence rather than expert opinion that this is a reasonable standard to set. We hypothesise that a range of factors have contributed to more effective use of clinical time: better referral guidance, use of the abbreviated pathway and triage, together with a planned approach to training and mentoring within all members of diagnosing teams and not just a few expert practitioners. There has been some debate about how we define the point of referral and we have followed the NAP-C (LeCouteur 2003) and AAA guidance (McKenzie et al. 2015) which take this date as the date referred for specialist assessment. It does not include the period of time pre-referral or during general developmental assessment (GDA) often conducted by community staff, which is necessary to establish whether an ASD assessment is indicated. Because this pathway was specifically focussed on aspects of the service specific to ASD, we did not calculate within service waiting times for GDA. During this year within service waits varied: for SLT (within 12 weeks) CCH (within 12 weeks). For CAMHS Choice appointments occur within 6 weeks and the wait between Choice appointment and allocation of a caseholder to start GDA was over 30 weeks. The high rates of positive ASD diagnoses (at 86% of referrals) suggest that the processes in place, support good use of clinical time and children not being unnecessarily sent to specialist teams when their difficulties are not explained by ASD.

### Waiting Times from Referral to Diagnosis Shared

There has been a steady downward trend in waiting times. During the consultation period, staff were alerted to the need to change practice and participated in discussions about how this could be done. It is possible that the increased focus on ASD service issues contributed to the reduction in the average duration of assessment from referral to diagnosis shared from 270 days in 2014 to 250 days in February 2015. After 12 months of pathway implementation the wait was 146.3 days (20.9 weeks) in January 2016 and the 10 month average for January to October 2016 was 122.5 days (17.5 weeks). In the current NHS climate in the UK, this statistically significant reduction in wait times (*t*(20) = 5.5, *p* = < 0.05) between 2015 and 2016 is a highly noteworthy finding. Through using evidence based clinical guidelines and literature evidence to write a pathway and adapt multi-disciplinary working practices, a large clinical service can establish a system for recording and monitoring the pathway and also maintain a more efficient service as evidenced by a reduction in duration of assessment over a two year period.

### Feasibility of the Pathway Implementation

*Monthly triage meetings* have been attended by a representative from CAMHS, CCH and SLT. While this is a demand on clinical time, the data reported and practitioner feedback suggest it is an important part of the solution for reasons outlined below. The meetings have: supported compliance with the pathway and increased the amount of joint working by allowing ongoing discussion and clarification between staff in different professional groups; provided quality assurance for the abbreviated pathway by reviewing information from community teams to ensure that a robust assessment is completed; provided a forum for discussion and action in complex cases; ensured that referrals were only accepted when there was adequate information available and therefore appointments were offered only when clinicians were in a position to use their time well (e.g. avoiding clinic appointments with no contextual assessment information); acted as a point of contact for referrers where they could discuss complexity and uncertainty and support effective decision making; where referrals bounced between agencies, the triage team could make a clear decision about who would complete the assessment and the team provided responsive management of staff absence or gaps in a particular part of the locality—e.g. staff or clinics were moved to avoid unfair waits and the triage team monitored referrals to prioritise where needed or to ensure cases remain under discussion until the pathway is complete. The NAP-C plan assumes that there is a wait between local and specialist teams (maximum 3 months) rather than the approach taken here, where there is triage after the general developmental assessment, to allocate to either a local (also referred to as abbreviated) or specialist (complex) process at the outset thus preventing the double within service wait.

*The abbreviated pathway* was applied on average in 11.3% of cases and in one locality was as high as 19%. In such cases, the first professionals who have completed the general developmental assessment prior to “referral” to triage for specialist assessment also complete the ASD assessment without involvement of another tier of practitioners. This has been supported by training and mentoring, so that the community paediatrician or CAMHS case worker (at the enhanced or expert level) completes the same early developmental history and contextual assessment questionnaires. A direct observation of the child is also an essential component. The SLT completes an ASD specific communication assessment and a member of the team gathers information from education colleagues, which may include a school observation. In the “abbreviated” pathway, full adherence to clinical guidelines is maintained and diagnosis is made through consideration of reports and observations in relation to DSM 5 criteria (American Psychiatric Association 2013). Families receive the same post-diagnostic information and an assessment report, detailing the process and outcome and any follow up referrals or assessments (e.g. genetics) are undertaken in the same manner. It is an aspiration to continue to increase the number of abbreviated assessments undertaken. The benefits to families of seeing fewer people and receiving more local care are only anecdotal at present and it would be helpful to review both the experiences of staff and families and the robustness of diagnoses made in this manner.

Future research to ensure that diagnoses made using the abbreviated pathway are robust, would be helpful. In the US context, concerns have been raised about children who have received “educational certification” of ASD diagnosis through school, which is used as an “abbreviated type” approach. In this study we were mindful of the need to evidence that health staff making the “abbreviated” diagnosis in a multi-disciplinary team have applied a robust process and reached a valid conclusion. Until we have more concrete research evidence, diagnostic robustness can be assumed if the team has: followed the local pathway guidance; followed national clinical guidelines and have the appropriate knowledge training as evidenced using the NHS ASD training framework.

### Limitations

One limitation of the current study was that data collection was undertaken by clinicians, leading to some inconsistency in recording before data cleaning. For example, different codes were used by staff to record the same thing or date format varied. This did not affect data accuracy.

A further limitation is the missing and incomplete data collected following implementation. The reasons for this have been explained within the results section and are further reported below. The baseline data was complete and for the data following implementation, there was no missing data for age at referral or triage outcomes.

The first type of incomplete data following implementation was for gender of referrals. These data were not collected in all localities. Gender was only recorded in one locality of the four, representing 43% of the dataset, with data from 143 cases. This sample size was considered worthy of report, although it is a subset of the entire sample. In future we would seek to collect this data for all cases.

The second type of incomplete data was for waiting times or diagnostic rate and is explained by the timing of the study. The study took place over a specified timeframe and 711 referrals were recorded as they came in. For all cases there was data for age of referral, number of referrals and triage outcome. Of these cases, 259 were completed and 453 referred cases were still undergoing assessment at the close of the timeframe. The later referred (453) cases had not progressed through the pathway to completion of assessment and were therefore not included in calculation of waiting times or diagnostic rate. Despite this limitation, we have explicitly reported the nature of the data for transparency. The sample size is large and therefore still considered important in this under-researched area.

The lack of inclusion of CAMHS cases in one locality, leading to a potential underestimate of the number of referrals, limits our ability to provide complete accuracy in referral number. Although there is strong evidence of the reliability of diagnoses made by experienced clinicians using evidence based guidelines (Charman and Baird 2002), this study does not provide secondary corroboration of diagnostic accuracy. The study does not report on individual ethnicity or socioeconomic status, which might be important variables to include in future studies.

## Conclusions and Implications for Practice

This study suggests that the service improvement approach followed, including the development of this single local pathway has been successful and has led to a significant reduction in waiting times. We strongly commend the benefits of good data collection and management to inform effective service planning. The intervention has been manageable and has been shown to have good feasibility within a large UK clinical service, receiving over 700 referrals per year and which shares universal challenges of recruitment and retention; succession planning and increasing demand for ASD diagnostic services without any increase in resource to meet the need. There is potential for short term resource to support the service improvement programme, resulting in sustainable changes in practice because this was not a small test of change but a highly evidence based model across a whole service. The gender ratio (2.7:1) suggests that more girls are being identified and the aspiration to support the recognition of girls may be having an effect. It would be helpful to continue to monitor this. Across all teams there is an aspiration that 25% of cases follow the abbreviated pathway, together with continued awareness raising to reduce the age of referral to support earlier diagnosis.
